# Deep brain stimulation electrodes may rotate after implantation—an animal study

**DOI:** 10.1007/s10143-020-01429-6

**Published:** 2020-10-30

**Authors:** Alexander Rau, H. Urbach, V. A. Coenen, K. Egger, P. C. Reinacher

**Affiliations:** 1 Department of Neuroradiology, Medical Center – University of Freiburg, Faculty of Medicine, University of Freiburg, Breisacher Str. 64, 79106 Freiburg, Germany; 2 Department of Stereotactic and Functional Neurosurgery, Medical Center – University of Freiburg, Faculty of Medicine, University of Freiburg, Freiburg, Germany; 3 Fraunhofer Institute for Laser Technology, Aachen, Germany

**Keywords:** Directional deep brain stimulation, DBS, Lead orientation, Rotational fluoroscopy

## Abstract

Directional deep brain stimulation (dDBS) electrodes allow to steer the electrical field in a specific direction. When implanted with torque, they may rotate for a certain time after implantation. The aim of this study was to evaluate whether and to which degree leads rotate in the first 24 h after implantation using a sheep brain model. dDBS electrodes were implanted in 14 sheep heads and 3D rotational fluoroscopy (3D-RF) scans were acquired to visualize the orientation of the electrode leads. Electrode leads were clockwise rotated just above the burr holes (180° *n* = 6, 360° *n* = 6, 2 controls) and 3D-RF scans were again acquired after 3, 6, 13, 17, and 24 h, respectively. One hundred eighty degree rotated electrodes showed an initial rotation of 83.5° (range: 35.4°–128.3°) and a rotation of 114.0° (range: 57°–162°) after 24 h. With 360° torsion, mean initial rotation was 201° (range: 3.3°–321.4°) and mean rotation after 24 h 215.7° (range 31.9°–334.7°), respectively. Direct postoperative imaging may not be accurate for determining the rotation of dDBS electrodes if torque is present.

## Introduction

Deep brain stimulation (DBS) is a well-established treatment for several disorders such as Parkinson’s disease, tremor, dystonia, and drug-resistant epilepsy. The latest generation of the so-called directional DBS electrodes (dDBS) contains multiple electrode contacts and allows to distribute the stimulation field between the lead segments, thus steering the electrical field in a pre-defined direction. This helps to avoid or reduce inadvertent events by stimulation of adjacent brain regions. To select stimulation parameters which allow for stimulation in a certain direction, the orientation of the dDBS lead in the individual patient brain needs to be known [1, 2].

Computed tomography, stereotactic x-ray, and recently 3D rotational fluoroscopy (3D-RF) are accurate imaging modalities to determine the rotation of dDBS [3–6]. They are considered to be very precise (deviation from true orientation: rotational fluoroscopy ± 2.44°; CT − 0.6 ± 1.5° (range: − 5.4 to 4.2°); flat-panel CT 5.4° ± 4.1° (range: 0.4°–11.9°); stereotactic x-ray 0.0° ± 5.0° (range: − 12° to 14°)).

Flexible dDBS electrodes are implanted into the brain using a stylet, where torsion of the electrode relative to the stylet is conceivable during implantation. The final orientation often shows deviations from 30° [7] up to 89° [8] from the intended rotation. This deviation may be caused by a brain shift due to CSF drainage, edema, or pneumocephalus [9, 10]; however, we hypothesized that it is due to an ongoing torque on the electrode after implantation, too. This ongoing torque may be accentuated, when an intraoperative correction of the rotation by turning the electrode at the skull level is performed [3, 7, 11]. If the electrode continues to rotate after implantation, direct postoperative imaging may be inadequate to obtain the dDBS rotation for stimulation settings [7, 8, 11].

In order to monitor the dDBS rotation for a certain time, we chose a sheep brain model, as it allows us to implant 10 cm of electrode length in brain tissue under relatively realistic conditions.

## Methods

**Fig. 1:**
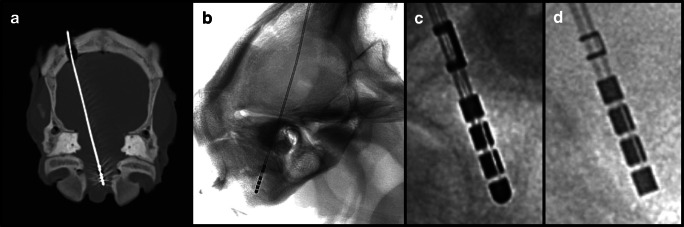
Imaging modalities in this modal trial. **a** CT scan after electrode implantation with maximum intensity projection showing a transparenchymal course of the electrode (Cartesia™). **b** Lateral projection (note the overlap of the meatus acusticus externus) of the 3D-RF with Cartesia™ implanted. **c** Excerpt from the 3D-RF, which depicts the so-called iron-sight sign of a Cartesia™ electrode. This occurs exactly orthogonal to the electrodes orientation—note the c-shaped lead marker. **d** “Iron-sight” sign of an Infinity™ electrode

dDBS electrodes were implanted in 15 sheep heads by a stereotactic neurosurgeon (16 years of experience), one electrode per head—nine times Cartesia™ (Boston Scientific, USA) and six times Infinity™ (Abbott Neuromodulation, Plano, TX, USA). Implantation was performed via a burr hole trephination and free hand as torsion free as possible. Directly after implantation, the electrodes were fixed with clamps anchored in the bone so that the electrodes could not rotate at this level. A helical CT scan was acquired immediately after implantation in order to check the position (XYZ coordinates) and whether the electrode runs through brain tissue over its entire length (Siemens Somatom Scope, Siemens Healthcare, Forchheim, Germany, voltage 130 kV, exposure, 90 mAs, pitch factor 0.33, tilt 0°, slice thickness 1.2 mm, helical mode). An example is given in Fig. 1a.

A 3D rotational fluoroscopy scan (3D-RF) was acquired in order to determine the dDBS rotation. This method has been described previously and selects one of 622 rotational projections, which shows the dDBS orientation clearly [3]. Afterwards, the fixation at the skull level was carefully released and the dDBS was rotated at the skull level by either 180° (*n* = 6) or 360° (*n* = 6) and the extracranial part was permanently fixed to the skull using the same technique, immediately after a second 3D-RF run was acquired. Another two dDBS electrodes were fixed after implantation without rotation and served as controls.

3D-RF runs were acquired after 3, 6, 13, 17, and 24 h and during this period, the sheep heads have carried a distance of 200 m to simulate the patient’s movement and stored at 37 °C.

3D-RF runs were acquired using a flat panel detector C arm (Allura Xper biplane FD20 20; Philips Healthcare, Best, the Netherlands). The 3D cerebral propeller scan acquires a total of 622 projections within 20.7 s covering a rotation range of 210° rotating from a left-sided position over the forehead to a right-sided lateral position (Fig. 1b).

The image data were evaluated using an image viewer capable of showing the acquisition angle (http://clinical.netforum.healthcare.philips.com/global/Explore/Clinical-News/MRI/Philips-DICOM-Viewer-download-version-R30- SP3). Initial rotation, rotation after applied torsion and after 3, 6, 13, 17, and 24 h, was evaluated accordingly [3].

## Results

In 14 sheep heads, implantation with a total parenchymal dDBS course was achieved. One sheep head was excluded because of accidental electrode dislocation.

In two controls, only slight deviations from the initial dDBS rotation were observed (1° and 4° after 24 h, respectively).

After 180° torsion, the mean initial rotational deviation was 83.5° (range: 35.4°–128.3°), and the mean rotational deviation after 24 h was 114.0° (range: 57°–162°), respectively. Thus, a mean rotational deviation of 30.5° (range: 14.1°–48.2°) occurred.

**Fig. 2:**
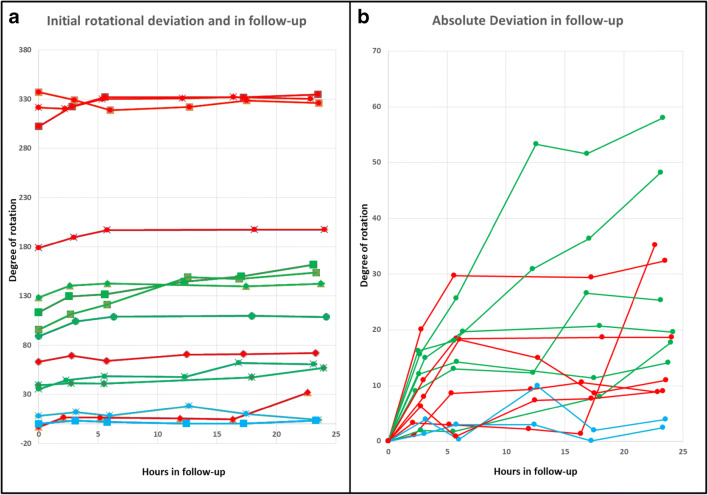
Rotational deviation of the implanted electrodes after applied torsion. 180° applied torsion depicted in GREEN, 360° in RED, and controls (0°) in BLUE. **a** Initial rotational deviation (time point 0 h) and in a follow-up. **b** Absolute deviation in relation to the rotation after applied torsion

The exact time course over 24 h is displayed in Table 1 and Fig. 2.

**Table 1:** Rotational deviation of the implanted electrodes after applied torsion

Sheep head	dDBS	Applied torsion	Initial rotational deviation	3 h	6 h	13 h	17 h	24 h	Rotational deviation after torsion until end of follow-up
1	Cartesia™	180°	95.7°	111.3°	121.3°	149.0°	147.3°	153.7°	58.0°
2	Cartesia™	180°	89.1°	104.1°	108.8°		109.8°	108.7°	19.6°
3	Cartesia™	180°	128.3°	140.4°	142.6°	139.7°	140.4°	142.4°	14.1°
4	Cartesia™	180°	113.4°	129.6°	131.4°	144.3°	149.8°	161.6°	48.2°
5	Cartesia™	180°	Exclusion	Exclusion	Exclusion	Exclusion	Exclusion	Exclusion	Exclusion
6	Infinity™	180°	35.4°	44.4°	48.4°	47.7°	61.4°	60.7°	25.3°
7	Infinity™	180°	39.3°	41.3°	41.0°		47.3°	57.0°	17.7°
	mean		83.5°					114.0°	30.5°
8	Cartesia™	360°	3.3°	6.6°	6.3°	5.5°	4.6°	31.9°	35.2°
9	Cartesia™	360°	302.3°	322.4°	332.0°		331.7°	334.7°	32.4°
10	Cartesia™	360°	337.0°	329.0°	318.7°		322°	328.4°	8.6°
11	Infinity™	360°	321.4°	320.3°	330.0°	330.7°	332°	330.3°	8.9°
12	Infinity™	360°	178.7°	189.7°	197.1°		197.4°	197.4°	18.7°
13	Infinity™	360°	63.1°	69.4°	64.0°	70.5°	70.8°	72.1°	9.0°
	mean	360°	201.0°						18.8°
14	Cartesia™	0°	8°	11.9°	8.9°	8.0°	10.0°	4.1°	3.9°
15	Infinity™	0°	− 3.2°	1.9°	0.2°		3.3°	0.8°	4.0°

With 360° torsion, mean initial rotation was 201° (range: 3.3°–321.4°), and mean rotational deviation after 24 h was 215.7° (range 31.9°–334.7°), respectively. Thus, a mean rotational deviation of 18.8° (range: 8.6°–35.2°) occurred.

## Discussion

When a dDBS electrode is implanted with torque or when the rotation is corrected intraoperatively by rotating it at the burr hole, it takes some time until the rotational torque is transferred to the level of contacts. It has been suggested that there is no rotation over time in leads that do not show rotational deviation during or right after the implantation [12], which might be true in dDBS electrodes implanted without torque. Dembek and colleagues have shown that there is no rotation over time after their first postoperative CT scan ~ 24–72 h after implantation [13]. We carried out this investigation to evaluate the immediate time course (< 25 h) of the rotational deviation at the contact level of dDBS electrodes implanted with torsion.

Interestingly, after the applied rotation, not the entire amount of torsion applied was transferred to the level of contacts as it already has been described in the literature that the torsion of the electrode at the skull level is not completely transferred to the level of contacts [3]. Overall, there was a mean transfer of approximately 60% of the applied torsion. It remains unclear whether this corresponds to the final rotation or whether there was further torque along the electrodes. The extent of rotation in the follow-up—after initial rotation—cannot be predicted as there were no differences between 180 and 360° rotated dDBS. Interestingly, after 360° applied torsion, mean rotational deviation was smaller than after 180° (18.8° vs. 30.5°), an explanation might be, that at some point, the static friction along the electrode was overcome. The time course is also unpredictable; however, deviations were larger within the first 6 h following implantation (Fig. 2).

As there are rotational deviations over a time course of at least 24 h, we consider immediate postoperative imaging as suboptimal for determining the dDBS rotation. This might also be true if the implantation is as torsion free as possible since the electrode itself is a rotationally flexible cable [8], thus torque might be present.

Imaging modalities to obtain the dDBS rotation should be both accurate and of low radiation dose.

We are aware of some limitations of this experiment: An intraoperative rotation of 180° or 360° is not realistic and was only chosen for this experiment. The sheep model only partially simulates the human condition. However, with respect to body temperature, dDBS length, and parenchymal course, the sheep model is likely the best animal model for this experiment. In vivo human studies are not allowed for this proof of concept study due to radiation exposure and one would not deliberately apply such torque to the leads in a human.

## Conclusion

In order to obtain the exact rotation of directional DBS electrodes, specific imaging should be acquired at the latest possible time point (e.g., on the day of discharge) but earliest 24 h after implantation. Furthermore, we suggest not to deliberately apply torque to dDBS electrodes during or after implantation.

## Data Availability

The datasets analyzed during the current study are available from the corresponding author on reasonable request.

## References

[1] Pollo C, Kaelin-Lang A, Oertel MF, Stieglitz L, Taub E, Fuhr P, Lozano AM, Raabe A, Schüpbach M (2014). Directional deep brain stimulation: an intraoperative double-blind pilot study. Brain..

[2] Steigerwald F, Müller L, Johannes S, Matthies C, Volkmann J (2016). Directional deep brain stimulation of the subthalamic nucleus: a pilot study using a novel neurostimulation device: horizontal current steering in DBS. Mov Disord.

[3] Reinacher PC, Krüger MT, Coenen VA, Shah M, Roelz R, Jenkner C, Egger K (2017). Determining the orientation of directional deep brain stimulation electrodes using 3D rotational fluoroscopy. AJNR Am J Neuroradiol.

[4] Sitz A, Hoevels M, Hellerbach A, Gierich A, Luyken K, Dembek TA, Klehr M, Wirths J, Visser-Vandewalle V, Treuer H (2017). Determining the orientation angle of directional leads for deep brain stimulation using computed tomography and digital x-ray imaging: a phantom study. Med Phys.

[5] Hunsche S, Neudorfer C, Majdoub FE, Maarouf M, Sauner D (2019). Determining the rotational orientation of directional deep brain stimulation leads employing flatpanel computed tomography, Oper. Neurosurg.

[6] Hellerbach A, Dembek TA, Hoevels M, Holz JA, Gierich A, Luyken K, Barbe MT, Wirths J, Visser-Vandewalle V, Treuer H (2018). DiODe: directional orientation detection of segmented deep brain stimulation leads: a sequential algorithm based on CT imaging. Stereotact Funct Neurosurg.

[7] Steigerwald F, Matthies C, Volkmann J (2019). Directional deep brain stimulation. Neurotherapeutics.

[8] Dembek TA, Hoevels M, Hellerbach A, Horn A, Petry-Schmelzer JN, Borggrefe J, Wirths J, Dafsari HS, Barbe MT, Visser-Vandewalle V, Treuer H (2019). Directional DBS leads show large deviations from their intended implantation orientation. Parkinsonism Relat Disord.

[9] Morishita T, Hilliard JD, Okun MS, Neal D, Nestor KA, Peace D, Hozouri AA, Davidson MR, Bova FJ, Sporrer JM, Oyama G, Foote KD (2017). Postoperative lead migration in deep brain stimulation surgery: incidence, risk factors, and clinical impact. PLoS One.

[10] van den Munckhof P, Contarino MF, Bour LJ, Speelman JD, de Bie RM, Schuurman PR (2010). Postoperative curving and upward displacement of deep brain stimulation electrodes caused by brain shift. Neurosurgery.

[11] Hartmann CJ, Fliegen S, Groiss SJ, Wojtecki L, Schnitzler A (2019). An update on best practice of deep brain stimulation in Parkinson’s disease. Ther Adv Neurol Disord.

[12] Krueger MT, Naseri Y, Cavalloni F, Reinacher PC, Kägi G, Weber J, Brogle D, Bozinov O, Hägele-Link S, Brugger F (2020) Do directional deep brain stimulation leads rotate after implantation? Acta Neurochir (Wien). 10.1007/s00701-020-04568-3 Online ahead of print 10.1007/s00701-020-04568-3 32915306

[13] Dembek T, Asendorf A, Wirths J, Barbe MT, Visser-Vandewalle V, Treuer H. Temporal stability of Lead orientation in directional deep brain stimulation. Preprint 10.1159/000510883 33049735

